# High-Temperature-Resistant Composite Lost Circulation Materials for Oil-Based Drilling Fluids: Preparation, Performance, and Synergistic Mechanism

**DOI:** 10.3390/molecules31111818

**Published:** 2026-05-25

**Authors:** Yue Gao, Cheng Ma, Xuan Qi, Hao Yan, Changbao Wang, Junfeng Zhang

**Affiliations:** 1 College of Chemical and Materials Engineering, Hainan Vocational University of Science and Technology, Haikou 571126, China; 2 School of Petrchemical Engineering, Liaoning Petrochemical University, Fushun 113001, China; 3 School of Chemistry and Chemical Engineering, Hainan University, Haikou 570228, China

**Keywords:** emulsion polymerization, nanopolymer, plugging–inhibitor, thermal stability, shale inhibition

## Abstract

Lost circulation in oil-based drilling fluids (OBDFs) under high-temperature conditions remains a significant challenge in deep and ultra-deep drilling. In this study, a high-temperature-resistant composite lost circulation material (LCM) was developed based on a synergistic strategy combining rigid bridging–consolidation and flexible embedding–filling. Rigid self-consolidating particles were prepared by coating skeleton materials with modified thermosetting resin, while flexible oil-absorbing resin was synthesized via suspension polymerization. The materials exhibited excellent lipophilicity, thermal stability, and structural integrity at 150 °C, with oil absorption capacity up to 3.43 g/g. The optimized composite LCM showed superior plugging performance, achieving compressive strengths above 11 MPa in white oil and 5 MPa in base mud at 150 °C. Effective sealing of 1–3 mm pore structures was obtained with leakage volumes below 10 mL, and fractured formations could be successfully consolidated. Mechanistically, rigid particles provide structural bridging, flexible resin enables pore filling via swelling, and modified resin(thermosetting resin chemically modified to achieve self-consolidation) enhances consolidation and micro-pore sealing, resulting in a dense and high-strength plugging layer. This work provides a promising approach for designing high-performance LCMs for OBDFs in high-temperature drilling environments.

## 1. Introduction

With the continuous growth of global energy demand, oil and gas exploration increasingly extends into deep, ultra-deep, and high-temperature formations. Wellbore instability, particularly in shale formations characterized by abundant nanopores, microfractures, and high clay content, remains a major challenge, often leading to lost circulation, stuck pipes, and increased operational costs [1,2,3,4,5]. Oil-based drilling fluids (OBDFs) are widely used due to their superior lubricity, shale inhibition, and high-temperature resistance compared to water-based systems [6,7,8]. However, the occurrence of lost circulation in OBDFs remains frequent, especially in deep shale formations with fractures, posing a need for effective plugging strategies [7,8].

Traditional plugging approaches, including bridging materials, oil-absorbing resins, polymer gels, and their composites, have been developed to mitigate fluid invasion. Bridging materials rely on rigid particles to form mechanical frameworks that block narrow channels, while fibers and platelets enhance pore filling [9,10,11]. Oil-absorbing and expandable polymers swell upon contact with oil, providing flexible filling but often exhibit limited pressure-bearing capacity and thermal stability [12,13,14]. Polymer gels and curable systems provide better residence and adaptability but are generally limited by low thermal tolerance or uncontrollable curing times [15,16,17,18,19,20]. Although composite and curable materials have improved performance, challenges remain in achieving both high-temperature stability and effective retention in complex pore-fracture networks [21,22].

Recent advances have focused on multifunctional lost circulation materials (LCMs) that integrate rigid bridging and flexible filling mechanisms. Gao et al. developed a high-temperature-resistant composite LCM combining self-consolidating rigid particles and oil-absorbing resin, achieving effective sealing of 1–3 mm pores at 150 °C with compressive strengths above 11 MPa in white oil and 5 MPa in base mud [23]. This strategy synergistically leverages mechanical bridging, swelling, and in-situ consolidation, enhancing thermal stability and sealing efficiency in high-temperature OBDF applications. Nevertheless, the optimization of component ratios, resin modification, and the elucidation of microscale synergistic mechanisms remain underexplored, leaving gaps in understanding the multiscale performance of high-temperature LCMs.

To address these gaps, this study develops a novel high-temperature-resistant LCM for OBDFs based on a “rigid bridging–consolidation + flexible embedding–filling” strategy. Self-consolidating rigid particles are coated with modified thermosetting resin to provide both mechanical bridging and in-situ consolidation, while a flexible oil-absorbing resin is synthesized via suspension polymerization to enable pore filling with high lipophilicity. The thermal stability, oil absorption, structural integrity, and plugging performance of the composite system are systematically evaluated, with particular attention to the synergistic mechanism underlying enhanced sealing efficiency. This work aims to provide a reference for the rational design of high-performance LCMs suitable for deep, high-temperature drilling environments.

## 2. Materials and Methods

### 2.1. Materials and Reagents

The skeleton materials used in this study were obtained from commercially available granular minerals. Modified resin was prepared in the laboratory and used as a coating and binding agent. Ethanol (analytical grade) was used as a solvent.

Styrene (St), isobutyl methacrylate (i-BMA), and octadecyl methacrylate (SMA) were used as monomers for resin synthesis. Divinylbenzene (DVB) served as the crosslinking agent, and benzoyl peroxide (BPO) was employed as the initiator. Polyvinyl alcohol (PVA) was used as the dispersant in the aqueous phase.Therefore, all drugs were provided by Sigma-Aldrich (St. Louis, MO, USA).

White oil and oil-based drilling fluid were used as wetting media during consolidation experiments. All reagents were of analytical grade and used without further purification.

### 2.2. Preparation of Lost Circulation Materials

#### 2.2.1. Preparation of Self-Consolidating Rigid Materials

The skeleton material was cleaned with deionized water and ethanol, dried at 105 °C for 2 h, and sieved to the target particle size (0.3–1.0 mm). The modified thermosetting resin (10–20 wt% based on skeleton material) was dissolved in ethanol (solid content −20 wt%) under stirring. The dried skeleton material was added to the resin solution and stirred for 30 min to ensure uniform coating. After filtration, the coated particles were dried at 80 °C for 6 h. The coated particles were then cured in an oven at 120–150 °C for 2–4 h to form a crosslinked resin layer on the surface, yielding rigid self-consolidating particles.

#### 2.2.2. Synthesis of Oil-Absorbing Resin

PVA was dissolved in deionized water (1.0–1.5 wt%) as the stabilizer. The monomers (SMA/i-BMA/St with a molar ratio of 2:2:1) were mixed and added dropwise into the aqueous phase under high-speed stirring (600–800 rpm) at 80–85 °C under nitrogen. After 30 min, AIBN (0.5–1.0 wt% of total monomer) was added to initiate the polymerization. The reaction was maintained at 80–85 °C for 3–5 h. The obtained resin beads were filtered, washed with hot water and ethanol, and dried at 60 °C to constant weight.

#### 2.2.3. Formulation of Composite Lost Circulation Materials

**Figure 1:**
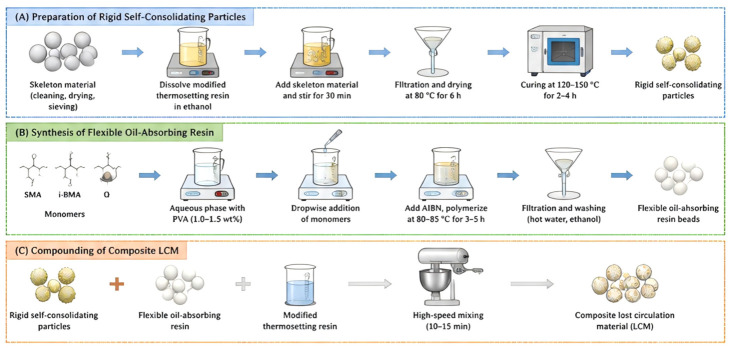
Schematic illustration of the preparation process of composite lost circulation material (LCM).

The rigid self-consolidating particles, flexible oil-absorbing resin, and modified thermosetting resin were mixed in a high-speed mixer. The typical mass ratio of the components was 50–70 wt% rigid particles, 20–40 wt% oil-absorbing resin, and 5–15 wt% modified resin. The mixture was blended for 10–15 min to ensure uniform distribution, yielding the final composite lost circulation material (LCM) (Figure 1).

### 2.3. Characterization Methods

The morphology of the prepared materials was observed using scanning electron microscopy (SEM). The particle size distribution was determined by standard sieving methods.

Thermal stability was evaluated using thermogravimetric analysis (TGA). The chemical structure of the synthesized resin was characterized by Fourier transform infrared spectroscopy (FTIR).

These characterization methods were used to analyze the structural features and thermal behavior of the materials.

### 2.4. Performance Evaluation

#### 2.4.1. Suspension Stability

The suspension stability of the composite lost circulation materials was evaluated by static settling tests in oil-based drilling fluid. A known amount of each material was added to the base fluid and stirred uniformly, then poured into a 200 mL graduated cylinder and allowed to settle at room temperature for 2 h. The mass of sedimented and suspended particles in the upper and lower halves of the cylinder was measured. The suspension rate (S, %) was calculated as:

S = (M_A_/(M_A_ + M_B_)) × 100%

where M_A_ is the mass of particles in the upper half and M_B_ is the mass in the lower half.

Table 1 shows the quantitative suspension data for Skeleton 1–3 at different organoclay contents (1%, 2%, 3%).

The data indicate that increasing organoclay content significantly improves suspension, with optimal stability achieved at 3 wt% organoclay for all skeleton types. Morphological observations further confirmed that Skeleton 1 and 2 maintained integrity after high-temperature aging, whereas Skeleton 3 showed partial degradation. These quantitative results provide direct evidence for the materials’ suspension performance and support their subsequent transport and retention in loss channels.

In this study, the term “Organic Soil Content” refers specifically to the mass fraction of organically modified clay added to the base drilling fluid to enhance suspension stability of the skeleton materials. It does not refer to rubber or other organic fillers. The organoclay serves as a dispersing and thickening agent, increasing the viscosity of the carrying fluid and promoting uniform suspension of the skeleton particles. By adjusting the organoclay content from 1 to 3 wt%, the suspension performance of Skeleton 1–3 was systematically evaluated, as shown in Table 1. Optimal suspension stability was achieved at 3 wt% organoclay for all skeleton types.

#### 2.4.2. High-Temperature Aging Resistance

The thermal stability of the materials was evaluated through high-temperature aging tests.

Samples were placed in sealed aging cells and subjected to elevated temperatures for a specified duration. After aging, the physical integrity and performance of the materials were assessed to determine their resistance to high-temperature conditions.

#### 2.4.3. Compressive Strength

The compressive strength of the consolidated samples was measured using a universal testing machine.

Cylindrical samples were subjected to axial compression at a constant loading rate until failure. The maximum load was recorded, and the compressive strength was calculated accordingly.

#### 2.4.4. Oil Absorption Capacity

The oil absorption capacity of the resin was determined using a gravimetric method.

A known mass of dry resin was immersed in oil for a specified time. After removing excess surface oil, the swollen resin was weighed. The oil absorption capacity was calculated based on the weight difference before and after absorption.

#### 2.4.5. Plugging Performance

The plugging performance of the composite materials was evaluated using a laboratory-scale plugging apparatus.

Simulated fractures or porous media were prepared, and the drilling fluid containing lost circulation materials was injected under controlled pressure conditions.

The pressure response and fluid loss were recorded to assess the plugging efficiency and sealing capability of the materials.

## 3. Results

### 3.1. Optimization and Performance of Self-Consolidating Rigid Materials

#### 3.1.1. Selection of Skeleton Materials and Suspension Stability

The selection of appropriate skeleton materials is critical for the formation of an effective plugging structure, as they primarily contribute to the bridging framework within loss channels. In this study, three types of skeleton materials, including two inorganic mineral materials (aggregate 1 and aggregate 2) and one organic plant-based material (aggregate 3), were evaluated in terms of suspension stability, morphology, and high-temperature resistance.

The suspension behavior of skeleton materials in oil-based drilling fluids directly affects their transport and distribution in the loss zone. By adjusting the content of organoclay in the base fluid, the suspension stability of different skeleton materials was systematically investigated. The results indicate that increasing the organoclay content significantly improves the suspension performance, and optimal suspension stability was achieved at 3 wt% organoclay (Table 2). Under this condition, all three materials exhibited “good” suspension behavior, ensuring uniform dispersion and minimizing sedimentation during pumping.

Table 2 Commentary (Skeleton 1–3 Explanation)

In Table 2, “Skeleton 1–3” represent three types of skeleton materials used for preparing self-consolidating rigid particles:

Skeleton 1 and Skeleton 2: Inorganic mineral materials with high thermal stability and mechanical strength, serving as the primary structural framework for bridging in loss channels.

Skeleton 3: Organic plant-based material with lower thermal stability, included primarily for comparison.

The parameters—apparent viscosity, plastic viscosity, and yield stress—reflect the suspension stability of each skeleton material in oil-based drilling fluids. The evaluation procedure is as follows:

A fixed amount of skeleton material was dispersed in the base fluid and stirred uniformly.

The mixture was poured into a graduated cylinder and left undisturbed at room temperature for 2 h.

The mass of sedimented and suspended particles in the upper and lower portions was measured.

Based on sedimentation behavior, Skeleton materials were classified as “Poor”, “Moderate”, or “Good”.

Morphological observations further confirmed that Skeleton 1–2 maintained integrity after high-temperature aging, whereas Skeleton 3 exhibited significant degradation, indicating that Skeleton 1–2 are more suitable for high-temperature lost circulation control.

**Figure 2:**
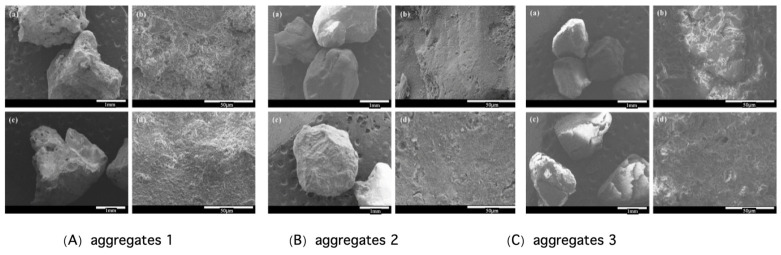
SEM images of skeleton materials before and after 210 °C/24 h aging ((**a**,**b**) are before aging, (**c**,**d**) are after aging).

Morphological observations reveal that all selected materials possess angular geometries, which are beneficial for mechanical interlocking and bridging within fracture channels. However, their thermal responses differ significantly. After aging at elevated temperatures, inorganic materials (aggregates 1 and 2) maintained their structural integrity with negligible morphological changes, whereas the organic material (aggregate 3) exhibited severe degradation, including surface cracking and structural loosening. This difference is attributed to the inherent thermal stability of mineral structures compared to the decomposition-prone nature of organic components (Figure 2).

These results demonstrate that inorganic skeleton materials are more suitable for high-temperature applications, providing stable structural support for subsequent consolidation.

#### 3.1.2. Thermal Stability and Degradation Behavior

To further quantify the thermal stability of the skeleton materials, particle size degradation and mass loss were evaluated after aging at different temperatures (150–210 °C). The results show that both aggregates 1 and 2 exhibit minimal particle size degradation and mass loss at 150 °C, indicating excellent thermal stability under typical downhole conditions. In contrast, aggregate 3 shows significant degradation at elevated temperatures, including increased particle size reduction and higher mass loss.

**Figure 3:**
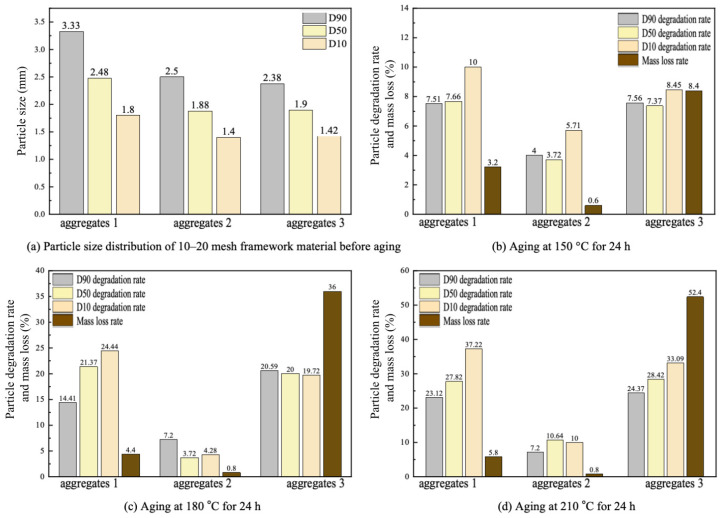
Particle size distribution, particle size degradation rate and mass loss rate of skeleton materials before and after aging of 10–20 mesh skeleton materials.

The superior thermal resistance of aggregates 1 and 2 can be attributed to their inorganic composition, which resists thermal decomposition. Conversely, the organic structure of aggregate 3 undergoes pyrolysis and carbonization, leading to structural weakening and reduced mechanical integrity. These findings confirm that aggregates 1 and 2 are more suitable as skeleton materials for high-temperature lost circulation control, particularly in formations exceeding 150 °C (Figure 3).

#### 3.1.3. Optimization of Thermosetting Resin and Curing Behavior

The thermosetting resin plays a key role in enabling the self-consolidation capability of the rigid materials. Three types of resins, including one modified resin and two commercially available resins, were compared in terms of thermal stability and consolidation performance.

**Figure 4:**
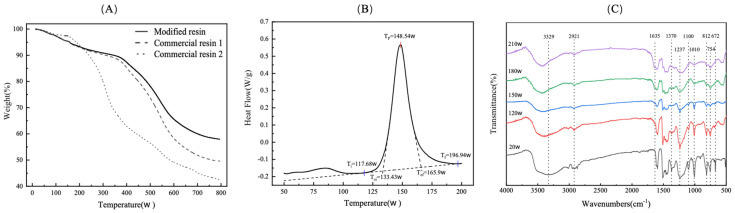
(**A**) Thermogravimetric curves of modified resin and commercially available resin 1 and 2; (**B**) DSC curve of modified resin; (**C**) infrared spectra of modified resin before and after curing at different temperatures.

Thermogravimetric analysis (TGA) shows that the modified resin exhibits superior thermal stability, with higher decomposition temperatures and a significantly higher char yield compared to commercial resins. This indicates a more stable molecular structure and enhanced resistance to thermal degradation (Figure 4A).

Differential scanning calorimetry (DSC) analysis reveals that the curing reaction of the modified resin occurs within a temperature range of approximately 133–166 °C, with the peak curing temperature around 150 °C. This temperature range is well aligned with typical downhole conditions, ensuring effective in-situ curing (Figure 4B).

Fourier transform infrared spectroscopy (FTIR) further elucidates the curing mechanism. The reduction of hydroxyl and ether functional groups, along with the formation of new carbonyl structures, confirms that condensation and crosslinking reactions occur during curing. These reactions lead to the formation of a three-dimensional network structure, which is responsible for the consolidation capability (Figure 4C).

In addition, compressive strength tests indicate that the modified resin-coated materials exhibit significantly higher consolidation strength than those coated with commercial resins, demonstrating its superior performance in both oil and drilling fluid systems (Table 3). Base slurry (oil-based drilling fluid system used as the carrier fluid for testing composite materials).

FTIR spectra of the synthesized oil-absorbing resin and several commercially available rubbers (natural rubber, nitrile rubber, and chloroprene rubber) were analyzed to elucidate functional group composition and potential chemical interactions. The oil-absorbing resin exhibits characteristic absorption bands at 1730 cm^−1^ (C=O stretching of ester groups), 1456 cm^−1^ (C–H bending), and 1160 cm^−1^ (C–O–C stretching), indicating the presence of ester and ether functionalities introduced by SMA, i-BMA, and St monomers. In comparison, natural rubber shows dominant C=C stretching at 1660 cm^−1^ and CH_2_ deformation at 1450 cm^−1^, while nitrile rubber displays a strong –C≡N stretching band at 2240 cm^−1^, and chloroprene rubber exhibits C–Cl stretching at 820 cm^−1^.

The unique ester and styrene-derived functionalities in the synthesized resin confer both lipophilicity and partial swelling capability, which are absent in conventional rubbers. Moreover, the crosslink density indicated by the broad absorption around 3400–3500 cm^−1^ (–OH groups from residual PVA) suggests moderate network formation, contributing to thermal stability. This comparison demonstrates that the synthesized oil-absorbing resin possesses a distinct chemical structure optimized for oil absorption and pore-filling in lost circulation applications, differing significantly from traditional rubber materials.

The modified thermosetting resin used as a coating and binding agent is based on a phenolic epoxy hybrid backbone, functionalized to enhance thermal stability and bonding performance. Reactive groups such as epoxide rings, hydroxyl groups, and methacrylate moieties are introduced via partial esterification and free radical copolymerization, providing sites for crosslinking during thermal consolidation. The modification ensures a controlled degree of crosslinking, promoting formation of a dense polymer network upon heating. The resin exhibits a molecular weight of approximately 15,000–25,000 g/mol and a functionality of 2.5–3.0 reactive sites per molecule. FTIR, DSC, and TG analyses confirm the presence of C=O, C–O–C, and –OH groups and demonstrate curing onset at ~120 °C and decomposition onset above 280 °C, indicating enhanced thermal stability. This modified resin coats the skeleton materials uniformly, enabling mechanical bridging, in-situ consolidation, and micro-pore sealing within the self-consolidating lost circulation material.

#### 3.1.4. Consolidation Mechanism of Self-Consolidating Materials

The self-consolidation behavior of the developed materials is primarily attributed to the thermal curing of the modified resin coated on the surface of skeleton particles. Under high-temperature conditions, the resin undergoes crosslinking reactions, forming a rigid three-dimensional network that binds adjacent particles together.

**Figure 5:**
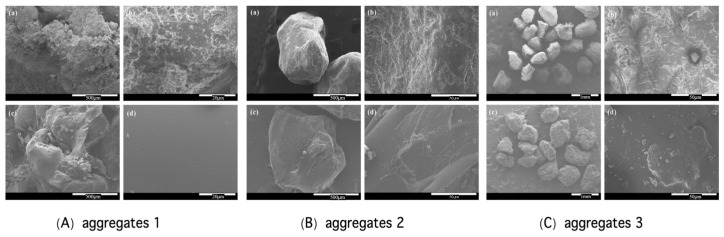
SEM images of the skeleton material before and after film coating ((**a**,**b**) are before film coating, (**c**,**d**) are after film coating).

Scanning electron microscopy (SEM) observations confirm that, after curing, distinct bonding points are formed between particles, resulting in a dense and continuous consolidated structure. Unlike conventional bridging materials, which rely solely on mechanical stacking, the self-consolidating materials achieve chemical bonding between particles, significantly enhancing structural integrity (Figure 5).

Therefore, the plugging mechanism of the rigid materials can be described as a combination of mechanical bridging and chemical consolidation. This dual mechanism effectively improves the retention capacity and resistance to fluid erosion in loss channels.

#### 3.1.5. Effect of Key Parameters on Compressive Strength

The compressive strength of the consolidated structure is a critical indicator of plugging stability and retention capacity. Several key parameters, including particle size, coating amount, curing temperature, and curing time, were systematically investigated.

**Figure 6:**
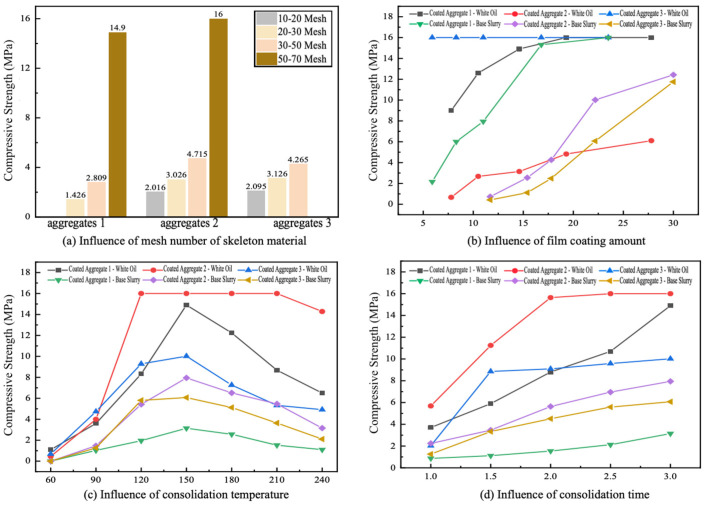
(**a**) Influence of mesh number of skeleton material on compressive strength of consolidations; (**b**) influence of film coating amount on compressive strength of consolidations; (**c**) influence of consolidation temperature on compressive strength of consolidations; (**d**) influence of consolidation time on compressive strength of consolidations.

The results show that smaller particle sizes lead to higher compressive strength due to the increased specific surface area, which enhances resin coverage and interparticle bonding. However, excessively fine particles may negatively affect permeability and transport behavior (Figure 6a).

The coating amount of resin also plays a crucial role. As the coating amount increases, the compressive strength initially increases due to improved bonding. However, beyond an optimal level, further increase in resin content leads to diminishing returns and increased material cost. Therefore, an optimal coating ratio must be determined to balance performance and efficiency (Figure 6b).

Curing temperature exhibits a typical “increase–decrease” trend in compressive strength. At low temperatures, insufficient curing results in weak bonding, while excessively high temperatures may degrade the resin network. The optimal curing temperature is around 150 °C, consistent with the DSC results (Figure 6c).

Similarly, curing time influences the degree of crosslinking. Adequate curing time ensures complete network formation, while excessive curing may not significantly improve strength but increases operational time (Figure 6d).

#### 3.1.6. High-Temperature Aging Resistance

The long-term stability of the consolidated structure under high-temperature conditions is essential for practical applications. Aging tests conducted at 150 °C for 48 h show that the consolidated samples retain considerable compressive strength, with values remaining above 2 MPa.

**Figure 7:**
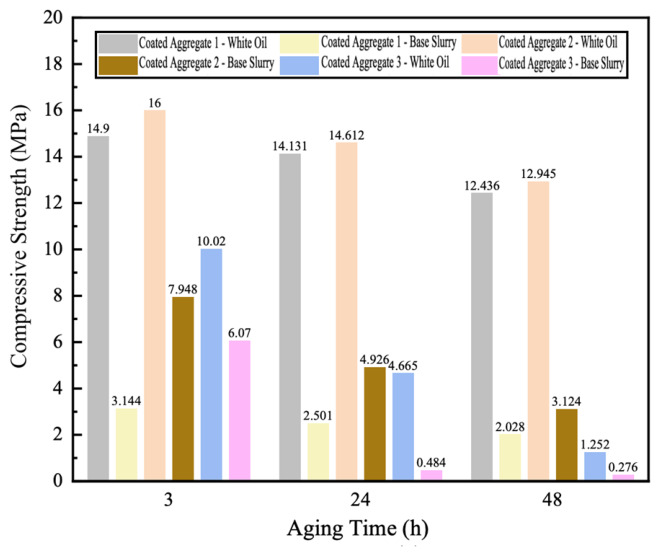
Influence of aging time on compressive strength of consolidations of self-consolidation lost circulation materials.

This result indicates that the self-consolidating materials possess excellent thermal stability and resistance to degradation under prolonged high-temperature exposure. The retained strength demonstrates that the resin network structure remains intact, ensuring sustained plugging performance in downhole environments (Figure 7).

In addition to TGA and high-temperature aging tests, dynamic mechanical analysis (DMA) was performed to quantify the evolution of mechanical properties with temperature. Cylindrical samples of self-consolidating composites were subjected to oscillatory stress in a single cantilever mode at a frequency of 1 Hz and a heating rate of 3 °C/min from 25 °C to 200 °C. The storage modulus (E′) and loss modulus (E″) were recorded to evaluate the material’s stiffness and energy dissipation capability.

Results show that the storage modulus of samples with inorganic skeletons (Skeleton 1–2) remains above 80% of the room-temperature value up to 150 °C, whereas Skeleton 3 exhibits a sharp decrease in E’ above 130 °C, consistent with observed particle degradation (Figure 7). Loss modulus peaks at ~160 °C indicate the onset of polymer softening, but the composite retains sufficient structural rigidity to maintain bridging and pore-filling function. These DMA results, combined with TGA and high-temperature aging, demonstrate that high char yield correlates with preserved mechanical integrity for the optimized composite LCM, validating its suitability for high-temperature downhole conditions.

Furthermore, the thermal evolution of compressive strength was evaluated by measuring strength at 25, 100, and 150 °C after 24 h thermal conditioning. Samples with Skeleton 1–2 retained more than 75–80% of their original compressive strength at 150 °C, confirming that mechanical integrity is maintained despite thermal exposure. This quantitative assessment bridges the gap between TGA-derived thermal decomposition and functional mechanical performance under operating temperatures.

### 3.2. Synthesis Optimization and Properties of Oil-Absorbing Resin

#### 3.2.1. Structural and Morphological Characterization

The chemical structure, morphology, and surface wettability of the synthesized oil-absorbing resin were systematically characterized to elucidate its role in the plugging system.

**Figure 8:**
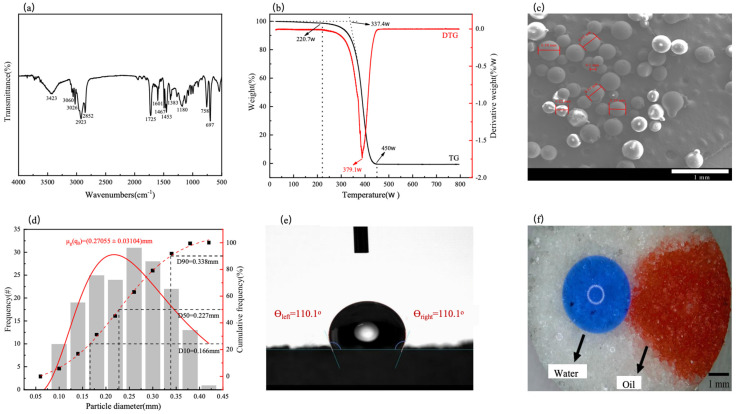
(**a**) Infrared spectrum of oil absorbing resin; (**b**) thermal weight loss curve and weight loss rate curve of oil absorbing resin; (**c**) SEM image of oil absorbing resin; (**d**) diameter distribution curve of oil absorbing resin; (**e**) the contact angle of the oil absorbing resin; (**f**) the presence of water and oil on its surface.

The FTIR spectra confirm the successful copolymerization of styrene (St), isobutyl methacrylate (i-BMA), and octadecyl methacrylate (SMA). Characteristic absorption peaks corresponding to aromatic C–H stretching, ester carbonyl (C=O), and long-chain alkyl groups are clearly observed, while the disappearance of C=C stretching vibrations indicates that the unsaturated double bonds participated in the polymerization reaction, leading to the formation of a three-dimensional crosslinked network structure (Figure 8a).

Thermogravimetric analysis reveals that the resin exhibits good thermal stability, with an initial decomposition temperature of approximately 220.7 °C. Below this temperature, only negligible mass loss is observed, which is mainly attributed to the evaporation of residual solvents and bound water. Significant decomposition occurs above 300 °C due to the breakdown of polymer side chains and network structure, indicating that the resin can maintain structural stability under typical downhole conditions (Figure 8b).

SEM observations show that the resin particles are predominantly spherical with smooth surfaces and without obvious structural defects. The particle size is mainly distributed in the range of 0.1–0.4 mm, with an average diameter of approximately 0.27 mm. Such morphology and size distribution are favorable for transport into formation pores and fractures (Figure 8c,d).

Contact angle measurements demonstrate that the resin possesses strong hydrophobicity and lipophilicity. The water contact angle is about 110°, indicating a hydrophobic surface, while oil droplets can be rapidly absorbed. This strong oil affinity ensures good compatibility with oil-based drilling fluids and facilitates efficient oil absorption and swelling behavior (Figure 8e,f).

#### 3.2.2. Optimization of Synthesis Parameters

The synthesis parameters of the oil-absorbing resin were systematically optimized with oil absorption capacity as the primary evaluation index.

The stirring speed significantly affects particle morphology and dispersion stability. At low stirring speeds, insufficient shear force leads to droplet aggregation and particle agglomeration, whereas excessively high speeds result in overly small particles and may damage the internal crosslinked structure. An optimal stirring speed of 400 rpm ensures uniform spherical particles with appropriate size and stability (Table 4).

**Figure 9:**
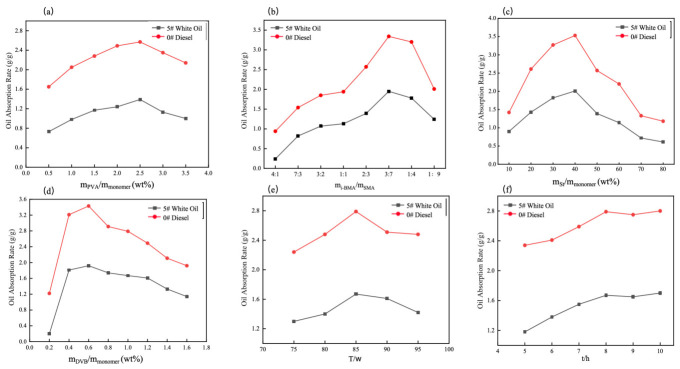
(**a**) Influence of PVA dosage on oil absorption performance of resin; (**b**) influence of SMA and i-BMA monomer ratio on oil absorption performance of resin; (**c**) influence of hard monomer dosage on oil absorption performance of resin; (**d**) influence of DVB dosage on oil absorption of resin; (**e**) influence of reaction temperature on oil absorption performance of resin; (**f**) influence of reaction time on oil absorption performance of resin.

The dispersant (PVA) content plays a key role in stabilizing the suspension system. When the dispersant content is insufficient, monomer droplets tend to coalesce, resulting in poor polymerization and low oil absorption capacity. In contrast, excessive dispersant increases system viscosity and residual surface coverage, hindering oil diffusion into the polymer network. The optimal dispersant dosage is 2.5 wt%, at which the resin exhibits maximum oil absorption performance (Figure 9a).

The monomer composition critically determines the balance between lipophilicity and network structure. Increasing the proportion of long-chain alkyl monomers (SMA) enhances oil affinity, but excessive SMA reduces the effective network volume and leads to particle adhesion. An optimal ratio of i-BMA to SMA of 2:3 achieves a balance between oil affinity and structural stability (Figure 9b).

The introduction of the rigid monomer styrene (St) improves the mechanical strength and thermal stability of the resin by incorporating benzene ring structures into the polymer backbone. However, excessive St restricts network expansion and reduces oil absorption capacity. The optimal St content is 40 wt% of total monomers (Figure 9c).

The crosslinking degree also plays a decisive role in swelling behavior. With increasing DVB content, the oil absorption capacity first increases and then decreases. At low crosslinking density, the network structure is insufficiently formed, whereas excessive crosslinking restricts network expansion. The optimal DVB dosage is 0.6 wt% (Figure 9d).

Additionally, reaction temperature and time influence polymerization efficiency. The optimal reaction conditions are 85 °C and 8 h, under which the polymerization proceeds sufficiently to form a stable three-dimensional network structure (Figure 9e,f).

To quantitatively evaluate the network structure and crosslinking density of the oil-absorbing resin, the Flory–Rehner swelling model was employed. The resin was swollen in 0# diesel oil and 5# white oil at room temperature until equilibrium. The volume swelling ratio Q was determined from the mass of resin before and after absorption.

The effective crosslinking density ν_e_ (mol·L^−1^) was calculated according to the Flory–Rehner equation:

V_e_ = −ln(1 − φ_r_) + φ_r_ + χφ_r_^2^/V_s_(φ_r_^1/3^ − φ_r_/2)

where φ_r_ is the polymer volume fraction in the swollen state, V_s_ is the molar volume of the solvent, and χ is the polymer–solvent interaction parameter.

The analysis revealed that increasing DVB content from 1 to 3 wt% led to a rise in crosslinking density from 0.018 to 0.035 mol·L^−1^, corresponding to decreased swelling ratios and increased network rigidity. Consequently, resins with higher DVB content exhibited slower initial oil uptake but improved mechanical stability during pumping and consolidation.

This quantitative analysis establishes a direct correlation between monomer composition, network rigidity, crosslink density, and observed oil absorption behavior, providing a rational explanation for the resin’s performance in lost circulation plugging applications.

#### 3.2.3. Oil Absorption Behavior and Thermal Stability

The oil absorption performance of the optimized resin was evaluated using diesel and white oil.

The results show that the resin exhibits high oil absorption capacity, reaching 3.43 g/g for diesel and 1.92 g/g for white oil at room temperature. The higher absorption capacity for diesel is attributed to its aromatic components, which interact more strongly with the benzene rings in the polymer structure.

The absorption process exhibits a typical swelling behavior. The initial absorption rate is relatively slow, which is beneficial for pumping and transportation in drilling operations. As time progresses, the absorption rate increases and reaches equilibrium within approximately 2 h.

Temperature significantly affects the absorption kinetics. With increasing temperature, the oil absorption rate accelerates due to enhanced molecular motion and reduced oil viscosity. However, the equilibrium absorption capacity shows only limited improvement, as the presence of rigid aromatic structures restricts excessive network expansion.

**Figure 10:**
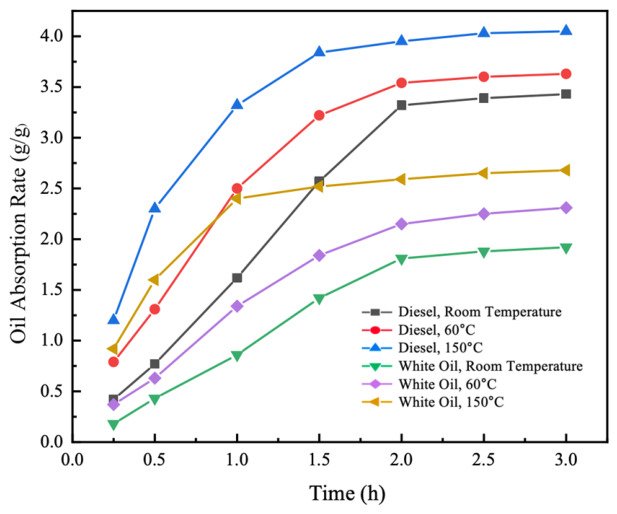
Curve of oil absorption rate of oil absorbing resin to 5# white oil and 0# diesel oil with time at different temperatures.

Furthermore, the resin maintains good structural integrity after oil absorption at 150 °C for 24 h, indicating excellent thermal resistance. The particles remain intact without collapse, demonstrating their suitability for high-temperature downhole environments (Figure 10).

Overall, the resin exhibits a combination of controlled swelling behavior, high oil absorption capacity, and strong thermal stability. These properties enable it to effectively fill pores and fractures and act as a flexible plugging component, complementing rigid bridging materials and enhancing the overall sealing performance of the composite system.

### 3.3. Performance Evaluation of Composite Lost Circulation Materials

#### 3.3.1. Optimization of Composite Formulation

**Figure 11:**
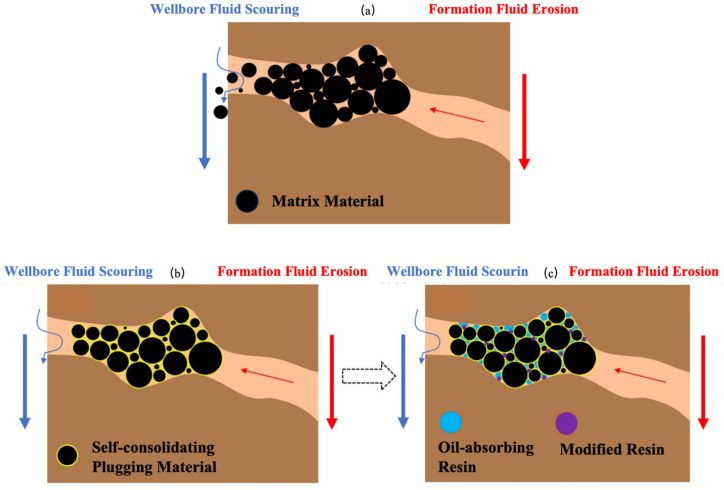
(**a**) Plugging mechanism of conventional bridging lost circulation material; (**b**) plugging mechanism of dual-function lost circulation material with “bridging” and “consolidation” effects; (**c**) synergistic Plugging Mechanism of Multiple Lost Circulation Materials.

To achieve effective plugging performance under high-temperature conditions, composite lost circulation materials (LCMs) were formulated based on the concept of “rigid bridging–flexible filling–chemical consolidation” (Figure 11).

The experimental results demonstrate that single-component systems exhibit limited plugging performance. Rigid self-consolidating materials can form an initial bridging framework; however, the absence of filling components results in large interparticle voids and poor sealing integrity. In contrast, oil-absorbing resin alone provides swelling and filling capability but lacks sufficient structural strength to withstand pressure.

Binary systems show improved performance due to partial synergistic effects. The combination of rigid materials and oil-absorbing resin enhances both bridging and filling; however, the stability of the plugging structure remains insufficient without effective consolidation under high-temperature conditions.

In contrast, ternary composite systems exhibit significantly enhanced performance. The rigid self-consolidating materials form the primary skeleton structure, the oil-absorbing resin expands to fill internal voids, and the modified resin enables thermal curing and interparticle bonding. As a result, a dense and integrated plugging layer is formed, in which particles are bonded together into a unified structure.

Therefore, the optimized formulation achieves a synergistic balance between structural support, deformability, and consolidation, which is essential for high-temperature lost circulation control.

#### 3.3.2. Plugging Performance Under Simulated Conditions

The plugging performance of the composite LCMs was evaluated using a sand-bed apparatus with steel ball packing to simulate formation pore structures under high-temperature conditions.

**Figure 12:**
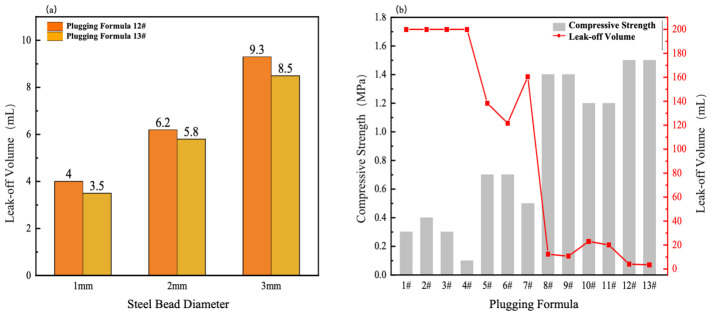
(**a**) Sealing effect of leakage plugging formula on steel ball beds with different particle sizes; (**b**) pressure bearing capacity and leakage of different plugging formulations.

The results show that, at 150 °C, the optimized composite system can effectively seal simulated pore structures formed by 1–3 mm steel balls, corresponding to pore sizes of approximately 0.15–1.24 mm (Figure 12a).

During the test, the plugging layer exhibits stable pressure-bearing capacity of up to 1.5 MPa, which is the maximum operating pressure of the experimental device. Meanwhile, the total fluid loss within 30 min remains below 10 mL, indicating excellent sealing efficiency (Figure 12b).

Observation of the formed plugging layer shows that the materials can penetrate into the steel ball bed and consolidate with the packing medium, forming a dense and continuous structure. The oil-absorbing resin fills the voids between rigid particles, further enhancing sealing integrity.

These results demonstrate that the composite system can rapidly form an effective sealing structure with strong pressure-bearing capability under high-temperature conditions.

### 3.4. Modified Resin Curing Mechanism

In addition to the previously described formulations, two further composite plugging formulations were evaluated: Formulation 12 and Formulation 13.

Formulation 12: Consists of self-consolidating rigid particles (skeleton material 1), oil-absorbing resin, and modified resin in a mass ratio of 50:30:20. This composition targets enhanced bridging and consolidation in 1–2 mm pores under high-temperature conditions.

Formulation 13: Includes self-consolidating rigid particles (skeleton material 2), oil-absorbing resin, modified resin, and an additional organoclay additive at a mass ratio of 45:30:20:5. This formula is designed to improve suspension stability and pore-filling efficiency in 1–3 mm fractures at 150 °C.

Both formulations were mixed thoroughly to ensure uniform distribution and subjected to the same performance evaluation as other formulations, including suspension stability, compressive strength, oil absorption, and plugging efficiency. Figure 12 presents the comparative performance of all formulations, including 12 and 13, demonstrating their effective pore sealing and consolidation behavior under simulated downhole conditions.

#### 3.4.1. Consolidation Strength in Different Media

The consolidation performance of the composite system is closely related to the properties of the self-consolidating materials.

Experimental results show that the compressive strength of the consolidated structures exceeds 10 MPa in white oil and remains above 3 MPa in base slurry systems, indicating strong consolidation capability in both simple and complex fluid environments.

After aging at 150 °C for 48 h, the compressive strength of the consolidated bodies formed by coated aggregates remains above 2 MPa in drilling fluid systems, demonstrating good resistance to high-temperature degradation and long-term structural stability.

Although the presence of drilling fluid additives slightly reduces the consolidation strength, the overall mechanical integrity remains sufficient to maintain effective plugging under downhole conditions.

The compressive strength of cylindrical consolidated samples (diameter 18 mm, length 50 mm) was measured using a universal testing machine (ST-5000N) at a constant axial displacement rate of 10 mm/min. For each formulation, five replicate samples (*n* = 5) were tested to ensure statistical reliability. The average compressive strength and standard deviation are reported in Table 5.

σ_c_ = F_max_/A (MPa)

where F_max_ is the maximum load at failure and A is the cross-sectional area.

Representative stress–strain curves (Figure 5) show a sharp failure for Skeleton 1 and 2, indicating predominantly brittle behavior, while Skeleton 3 exhibits a slightly extended plateau before fracture, indicative of semi-brittle behavior. These observations provide insights into the mechanical reliability and fracture resistance of the consolidated materials under simulated downhole conditions.

The inclusion of statistical treatment (mean ± SD, *n* = 5) and stress–strain behavior ensures the reproducibility and reliability of the reported mechanical data, providing a quantitative basis for evaluating plugging stability.

#### 3.4.2. Adaptability to Different Pore and Fracture Structures

The adaptability of the composite LCMs to different pore structures was evaluated based on particle size matching and bridging behavior.

The particle size distribution of the composite materials ranges from 0.22 to 0.6 mm, which is well matched with the simulated pore sizes (0.15–1.24 mm). The system satisfies the “two-thirds bridging rule”, ensuring effective bridging and packing within formation pores (Figure 12a).

Rigid particles with different sizes form a multi-scale bridging framework, while the oil-absorbing resin expands to fill residual voids that cannot be bridged by larger particles. This combination significantly improves packing density and reduces permeability within the plugging layer.

Therefore, the composite system exhibits strong adaptability to heterogeneous pore structures and can effectively seal both relatively large pores and smaller pore channels.

The microstructure of the composite lost circulation materials was further analyzed to elucidate the interactions between inorganic skeleton particles and polymeric resins. SEM observations reveal that the modified resin uniformly coats the surface of skeleton particles, forming a continuous thin layer (~1–3 µm), as evidenced by the high-magnification images of coated aggregates (Figure 3).

Quantitative evaluation of the coating amount (T%) demonstrates that Skeleton 1 and 2 achieve a resin coverage of 18–22 wt%, while Skeleton 3 reaches 14 wt% (Table 5). After thermal aging at 150 °C for 48 h, the inorganic aggregates maintain their coated layer integrity, whereas the organic aggregate exhibits partial resin detachment and surface microcracks, indicating weaker interface adhesion.

These observations suggest that the interface between resin and inorganic skeleton is predominantly physical adsorption enhanced by mechanical interlocking, while chemical bonding may occur via hydroxyl or silanol interactions at the particle surface. Although direct chemical characterization of the interface is challenging, the combination of SEM morphology, coating weight, and thermal aging resistance provides indirect evidence of strong interfacial adhesion for Skeleton 1–2. Pore-filling efficiency is high, with microvoids largely occupied by resin, and phase distribution appears homogeneous at the micron scale, ensuring effective bridging and consolidation within the composite (Figure 3).

Overall, these results indicate that interface adhesion and uniform resin coverage are critical for mechanical stability, high-temperature resistance, and pore-sealing performance of the composite lost circulation materials.

## 4. Conclusions

In this study, a high-temperature-resistant lost circulation material system for oil-based drilling fluids was developed by combining self-consolidating rigid materials, oil-absorbing resin, and modified thermosetting resin. The physicochemical properties, thermal stability, and plugging performance of the system were systematically evaluated, and its plugging mechanism was analyzed.

The results show that the self-consolidating rigid materials exhibit good thermal stability and mechanical strength, maintaining structural integrity under high-temperature conditions and providing an effective bridging framework. The oil-absorbing resin demonstrates strong lipophilicity and controlled swelling behavior, enabling effective filling of pores and fractures after oil absorption. The introduction of modified resin further enhances consolidation through thermal curing, resulting in a stable and compact plugging structure.

Performance evaluation indicates that the composite system possesses excellent plugging efficiency. The optimized formulation can effectively seal pore structures of 1–3 mm, with fluid loss lower than 10 mL. Meanwhile, the compressive strength of the consolidated body can reach more than 11 MPa in white oil and more than 5 MPa in base slurry at 150 °C, demonstrating strong consolidation ability under different media conditions.

The plugging mechanism involves the synergistic effect of rigid bridging, resin swelling, and thermal curing consolidation. Specifically, rigid materials provide structural support, the oil-absorbing resin fills and expands within pore spaces, and the modified resin enhances bonding and fills smaller voids, jointly forming a dense plugging layer.

Overall, the developed composite lost circulation material provides an effective solution for lost circulation control in high-temperature formations and exhibits promising potential for application in oil-based drilling systems.

## Figures and Tables

**Table 1:** Quantitative Suspension Stability of Skeleton Materials.

Organic Soil Content (%)	Skeleton 1	Skeleton 2	Skeleton 3
1	12	7	5
2	22.5	14	8.5
3	30	18	15

**Table 2:** Influence of Organic Soil Addition on Suspension Stability of Skeleton Materials.

Organic Soil Content (%)	Apparent Viscosity (mPa·s)	Plastic Viscosity (mPa·s)	Yield Stress (Pa)	Skeleton 1	Skeleton 2	Skeleton 3
1	12	7	5	Poor	Poor	Moderate
2	22.5	14	8.5	Good	Moderate	Good
3	30	18	15	Good	Good	Good

**Table 3:** Compressive strength of Aggregate 1 consolidations after coating with different resins.

Resin Type	m (Thermosetting Resin):m (Aggregate 1)	Dispersion System	Compressive Strength/MPa
Modified resin	1:4	White oil	14.900
		Base slurry	3.144
Commercial resin 1	1:4	White oil	8.192
		Base slurry	0
Commercial resin 2	1:4	White oil	2.627
		Base slurry	0

**Table 4:** Influence of stirring rate on the morphology of oil-absorbing resin.

Stirring Speed/rpm	System Dispersion	Polymerization Process	Resin Particle Appearance
200	Poor	Agglomeration, caking	Large particle size, severe caking
300	Good	No agglomeration	Relatively large particle size, non-uniform
400	Good	No agglomeration	Moderate particle size, relatively uniform
500	Good	No agglomeration	Excessively small particle size, uniform

**Table 5:** Compressive strength of consolidated samples at 150 °C.

Formulation	Skeleton Type	Compressive Strength (MPa, Mean ± SD, *n* = 5)	Observed Failure Mode
F1	Skeleton 1	11.2 ± 0.8	Brittle
F2	Skeleton 2	10.5 ± 0.7	Brittle
F3	Skeleton 3	7.8 ± 1.0	Semi-brittle

## Data Availability

The data that support the findings of this study are available from the corresponding authors due to privacy.
